# Virtual reality as a teaching method for resuscitation training in undergraduate first year medical students: a randomized controlled trial

**DOI:** 10.1186/s13049-021-00836-y

**Published:** 2021-02-01

**Authors:** Malte Issleib, Alina Kromer, Hans O. Pinnschmidt, Christoph Süss-Havemann, Jens C. Kubitz

**Affiliations:** 1 Department of Anaesthesiology, University Medical Center Hamburg-Eppendorf, Martini-Str. 52, 20246 Hamburg, Germany; 2 Department of Medical Biometry and Epidemiology, University Medical Center Hamburg-Eppendorf, Martini-Str. 52, 20246 Hamburg, Germany; 3 Department of Anaesthesiology and Intensive Care Medicine, Paracelsus Medical University Nuremberg, Breslauer Straße 201, 90471 Nuremberg, Germany

**Keywords:** Resuscitation, Basic life support training, Virtual reality, Medical school

## Abstract

**Background:**

Virtual reality is an innovative technology for medical education associated with high empirical realism.

Therefore, this study compares a conventional cardiopulmonary resuscitation (CPR) training with a Virtual Reality (VR) training aiming to demonstrate: (a) non-inferiority of the VR intervention in respect of no flow time and (b) superiority in respect of subjective learning gain.

**Methods:**

In this controlled randomized study first year, undergraduate students were allocated in the intervention group and the control group. Fifty-six participants were randomized to the intervention group and 104 participants to the control group. The intervention group received an individual 35-min VR Basic Life Support (BLS) course and a basic skill training. The control group took part in a “classic” BLS-course with a seminar and a basic skill training.

The groups were compared in respect of no flow time in a final 3-min BLS examination (primary outcome) and their learning gain (secondary outcome) assessed with a comparative self-assessment (CSA) using a questionnaire at the beginning and the end of the course. Data analysis was performed with a general linear fixed effects model.

**Results:**

The no flow time was significantly shorter in the control group (Mean values: control group 82 s vs. intervention group 93 s; *p* = 0.000). In the CSA participants of the intervention group had a higher learning gain in 6 out of 11 items of the questionnaire (*p* < 0.05).

**Conclusion:**

A “classic” BLS-course with a seminar and training seems superior to VR in teaching technical skills. However, overall learning gain was higher with VR. Future BLS course-formats should consider the integration of VR technique into the classic CPR training or vice versa, to use the advantage of both teaching techniques.

**Supplementary Information:**

The online version contains supplementary material available at 10.1186/s13049-021-00836-y.

## Introduction

The most important determinant of survival from sudden cardiac arrest is the presence of a trained lay rescuer who is ready, willing, and able to act [1]. It is particularly important to directly initiate chest compressions, as the chance of survival is significantly improved if the no flow time is kept as low as possible [2]. This emphasizes the relevance of good Basic Life Support (BLS) training not only for health care professionals, but also for a major part of the population. Classic instructor-led hands-on training in groups using a training mannequin is the most common training method worldwide [3]. According to the current resuscitation guidelines from 2015 of the European Resuscitation Council, BLS skills decrease quickly within 6–9 months, if cardiopulmonary resuscitation (CPR) is not regularly performed [1]. In this context, it is important to search for new training methods.

Virtual Reality (VR) is a forward-looking innovative simulation technique. It is a computer-generated simulation of the real or imaginary world that offers real time interaction opportunities [4]. Special hardware, such as VR glasses and controller, allows the user to experience surroundings and situations nearly as if he was really there [5]. Because of this high level of immersion, VR is an interesting and promising new way of teaching in a medical context, and its use is quickly expanding [6]. The user feels like a real actor and not like a spectator. Every conceivable scenario can be implemented in a virtual world via the software and offers the user numerous possibilities. VR may support both undergraduate and postgraduate medical education. It offers a better access to contextual and factual relationships to the user [7]. The learning experience in the virtual world is reproducible and controllable, but also flexible, which makes it an interesting medium [8]. So far, VR has been tested in different medical fields such as laparoscopic and orthopedic surgery. According to two systematic reviews a training supported with VR can improve technical skills in orthopedic surgery [9], and it can decrease operation time and improve operative performance [10]. Apart from technical skills, VR has the potential to increase the overall learning gain [11].

This controlled randomized trial was to investigate the hypothesis that BLS training in VR has no inferiority in respect of no flow time compared to standard BLS training (primary outcome). Further, it was to demonstrate that a high immersion and positive learning gain is achieved, if VR is used for BLS training (secondary outcome).

## Material and methods

This study was performed at the Medical Faculty of the University of Hamburg from January 7th until February 25th, 2019. In total 160 undergraduate students in their first year voluntarily participated in this study.

### Study design

In this randomized controlled study, first year medical students having their course in BLS were enrolled. As this was no study on humans, no formal ethical approval was required according to the local ethics committee.

At the beginning of each course the participants were randomized into the intervention group or control group. Furthermore, the participants received a study information form, a data protection form, a consent form and two questionnaires to evaluate the learning gain. The intervention group went through the BLS course in virtual reality, while the control group took part in the standard training. Both courses had the same learning objectives as described in the European Resuscitation Council (ERC) Guidelines [12]. At the end of the training, all participants performed a 3-min practical test. This test was done with the Leardal® Mannequin both groups were made familiar with in the previous course.

### Participants

The participants were undergraduate students in their first year. There were no exclusion criteria.

### Intervention

The participants were guided through the VR-BLS course individually. They went through a short teaching module on how to use the VR technology, which was directly followed by the VR-BLS course. The technical aspects of BLS were integrated into the VR module as described below. The technique of bag-mask-ventilation could not be implemented sufficiently in the VR module. For this reason, the participants received a demonstration and short training session of bag-mask-ventilation under supervision after the VR module. The VR-BLS course was a standardized 35 min test after which the participants were asked to perform the final test without further training.

#### Virtual reality module

The VR software was developed in cooperation between *Universitätsklinikum Hamburg Eppendorf* and *VIREED.*^1^

**Fig. 1:**
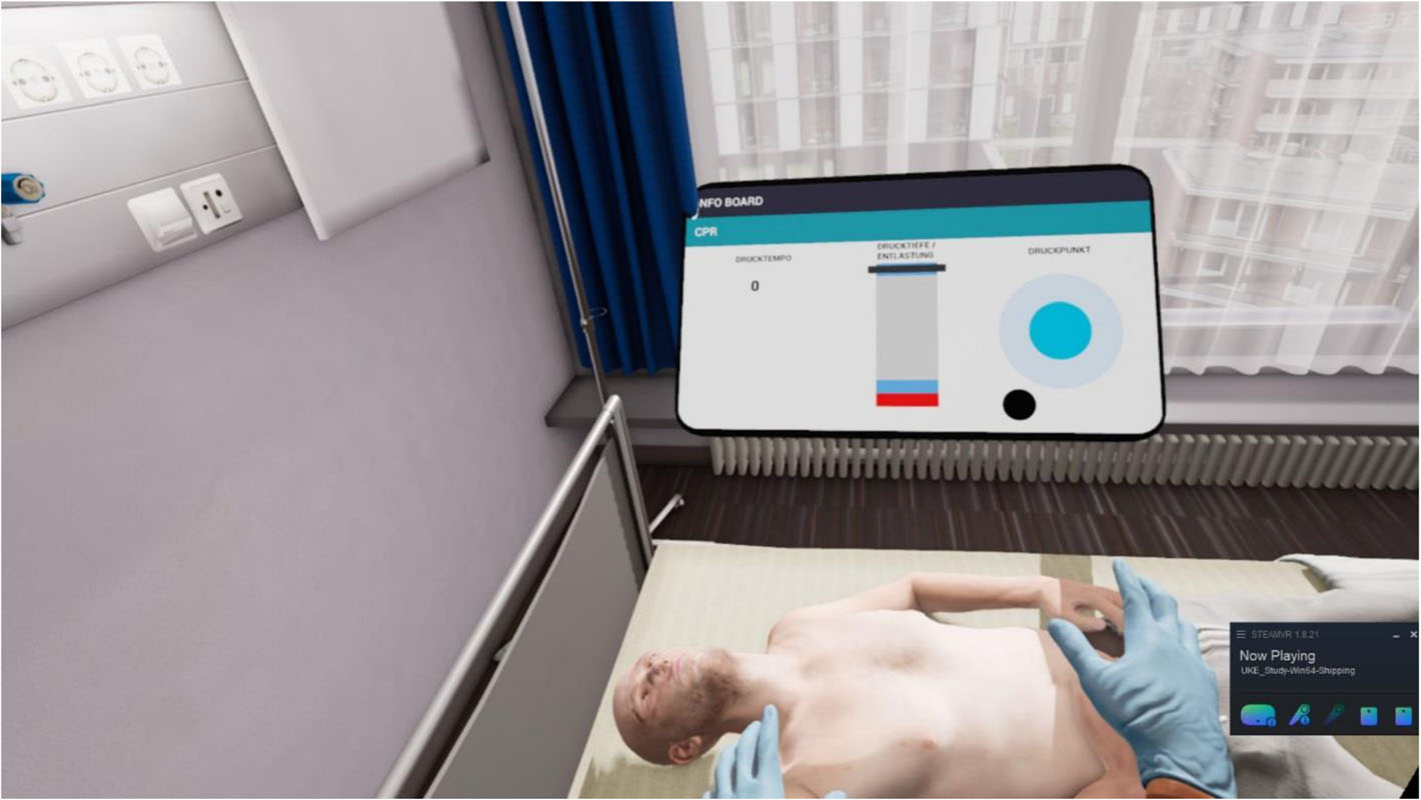
Visual Feedback Monitor for the quality of chest compressions in the VR Module

**Fig. 2:**
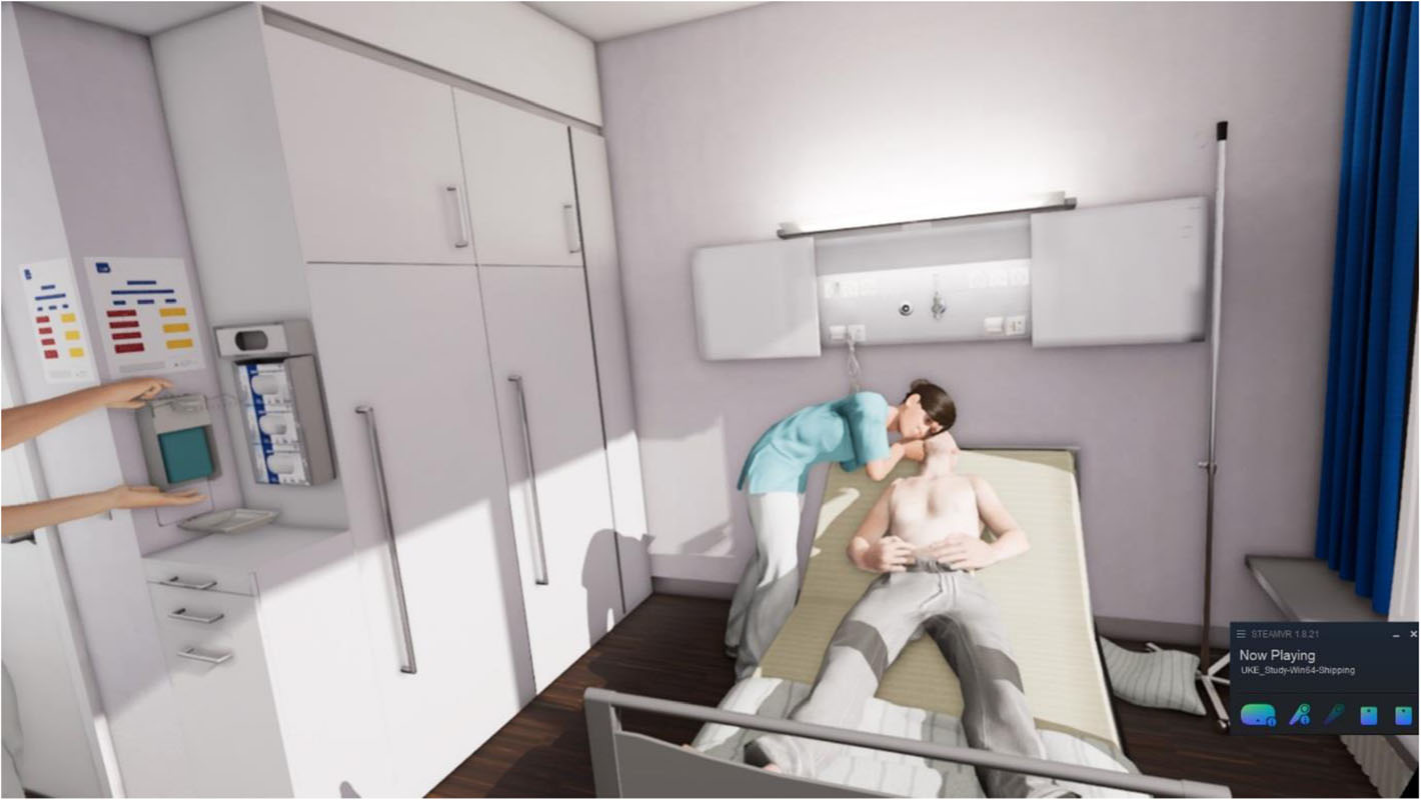
BLS real time scenario

The VR-BLS course is divided into two parts. In the first part, a correct BLS scenario is demonstrated to the participant over several steps with involving him actively in the process. The second part of the VR-BLS course focuses on the technique of chest compressions. The user performs chest compressions on the Leardal® QCPR Mannequin, which is connected to the VR system. This mannequin has the same torso and haptics as the one used in the control group and in the practical test. The user gets a visual feedback on the quality of chest compressions. The second part ends with a real-time scenario, where the user provides BLS without assistance (Figs. 1 and 2).

### Overview: the steps of the VR-course (part 1)

#### Step 1

Brief introduction to the software and hardware. Demonstration of all possibilities of interaction with the virtual world by a virtual professor.

#### Step 2

User as passive viewer in a hospital room. He is a spectator of a cardiac arrest, which is followed by BLS provided by clinical staff.

#### Step 3

User as passive viewer. The scenario is repeated. Each step is explained by the virtual professor.

#### Step 4

User as active BLS provider. Scenario is repeated, and the user carries out every step of the BLS independently. The scenario can only be continued, if all steps have been carried out.

### Control

The control group took part in the regular BLS course. This included a 45-min lecture on the background and technique of BLS, followed by a 1 h practical training using the Laerdal® QCPR Mannequin and the Laerdal® Skill Reporter Software under direct supervision of the lecturers. The ratio of learners to mannequins was 4:1. Participants could schedule the training themselves and, if necessary, repeat exercises within the time frame of 1 h.

### Outcomes

Primary outcome parameter was the no flow time, which was measured in a final 3-min practical examination. The examination was performed using a Leardal® QCPR Mannequin and the Leardal® Skill Reporter Software (Laerdal, Stavanger, Norway).

Secondary outcome was the learning gain, which was measured using a comparative self-assessment (CSA). The participants had to fill out a modification of a questionnaire originally developed and evaluated by Raupach et al. [13]. (CSA Questionnaire, Supplement 1). On a Likert scale from 1 to 6 (1 = mostly applies, 6 = mostly does not apply), the participants documented their self-assessment prior to, and after the intervention for 11 questions concerning BLS. The learning gain in points and in percent was calculated with a common formula to evaluate learning gain for each of the 11 questions in the CSA questionnaire: [13].

$$ \mathrm{CSA}\kern0.5em \mathrm{gain}\left(\%\right)=\frac{\mu_{\mathrm{pre}}\hbox{-} {\mu}_{\mathrm{post}}}{\mu_{\mathrm{pre}}\hbox{-} 1}\times 100 $$

All participants, who rated themselves the best possible at the beginning (1 = mostly apply) were excluded from the calculation of the percentages, because no learning gain was possible.

Tertiary outcome was the user-friendliness of the VR module. Therefore, the participants in the intervention group evaluated their experiences in a questionnaire called the system usability score (SUS, Supplement 2) [14]. The user-friendliness was rated on a 5-Point–Likert-Scale (1 = totally agree to 5 = totally disagree).

### Randomization

The randomization was performed by drawing lots. In each course, three lots were distributed for the intervention group and a different number of lots for the control group. For the length of the course, the number of participants in the intervention group had to be limited to three participants. Each student was given the same chance to be allocated to one or the other group. If demographic data had differed significantly, for example in respect of previous CPR experience, a matching would have been performed. Therefore, a larger number of participants was allowed for the control group.

### Statistics

Histograms of data distributions of dependent variables were visually examined and variances across categories of grouping variables were computed and assessed for homogeneity. Group differences regarding participants age were tested by t-test, group differences regarding distribution of sex, previous CPR experience and previous VR experience were tested by χ^2^ tests. A general linear fixed effects model was fitted to data of the dependent variable: No Flow Time in seconds assuming intervention group, age, sex, previous CPR experience, previous VR experience, Type of admission to the course and the interaction term intervention group x previous CPR experience as fixed effects. For the dependent variables CSA-difference and CSA difference measuring percentage gain, a general linear mixed effects model was applied, considering each participant as a random effect and each of the 11 CSA items of every participant as repeated measures. Fixed effects for the dependent variable CSA-difference were intervention group, CSA item, CSA pre-rating, age, sex, previous CPR experience, previous VR experience, type of admission to the course of the study as well as the interaction terms for intervention group x item and CSA pre-rating x item. For the dependent variable CSA difference measuring percentage gain, the same fixed effects were assumed except for the interaction term CSA pre-rating x item. Model-estimated marginal means with 95% confidence intervals were computed and pairwise group comparisons were done. Odds ratios and their 95% confidence intervals and *p* values were examined. All modelling work was done employing the GENLINMIXED routine of IBM SPSS 26 version.

## Results

Demographic data of the 160 students enrolled in this study is presented in Table 1. There were no significant differences between the two groups in respect of age, sex and previous experience with CPR and VR. Fifty-six participants were randomized to the intervention group and 104 participants were assigned to the control group.

**Table 1:**
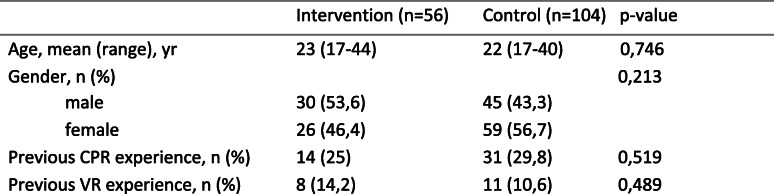
Demographic Data

**Fig. 3:**
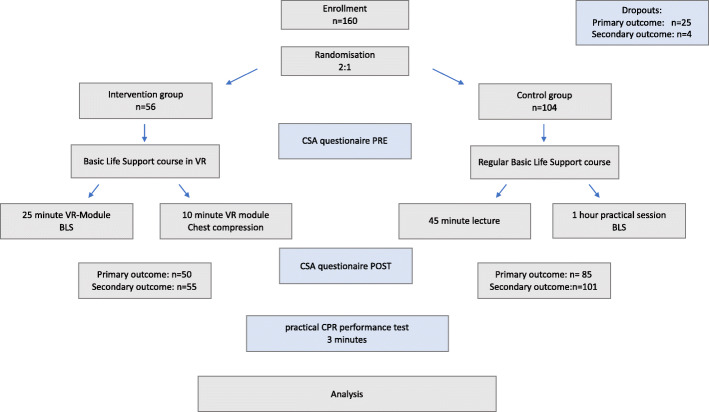
Flowchart of the study design

Complete data sets were obtained in 135 students for the primary outcome (no flow time) and in 156 for the secondary outcome (subjective learning gain). For the no flow time 19 participants in the control and 6 participants in the intervention group were excluded due to technical reasons. The questionnaires for the subjective learning gain were not completely filled out by 4 participants (intervention group *n* = 1, control group *n* = 3) (Fig. 3).

### Primary outcome: no flow time

The no flow time was significantly lower in the control group compared to the intervention group (control group mean: 82.031 s; intervention group mean: 92.963 s; *p* = 0.000), differing by 12%.

### Secondary outcome: CSA – subjective learning gain

**Fig. 4:**
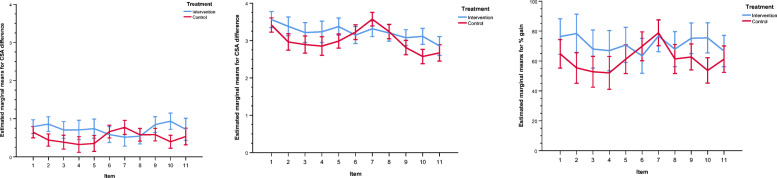
Graph of the differences of the CSA learning gain for the different items. The items 1–11 are plotted on the x-axis, and the estimated marginal mean difference in the CSA from pre- to post-intervention is plotted on the y-axis

**Fig. 5:**
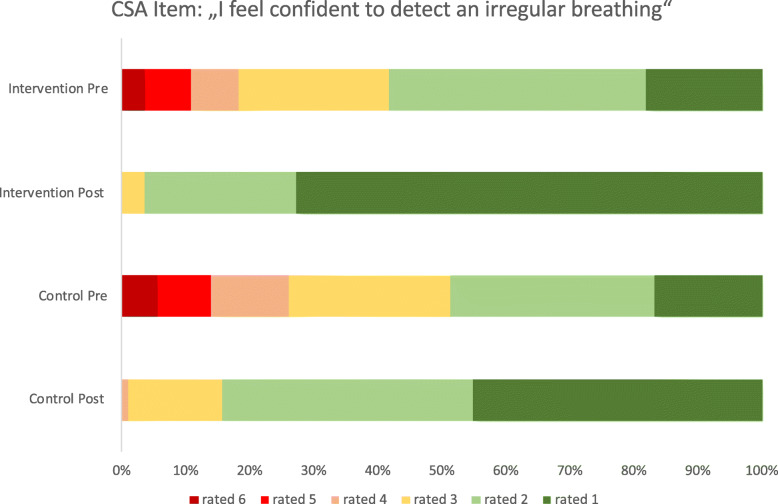
Descriptive analysis of learning gain pre and post interventional, graphics show the distribution of how confident the participants feel about the different advertised situations pre and post interventional. The evaluation offered 6 options from 1 = completely applies, 2 = mostly applies, 3 = still applies, 4 = rather does not apply, 5 = mostly does not apply, 6 = does not apply at all

**Fig. 6:**
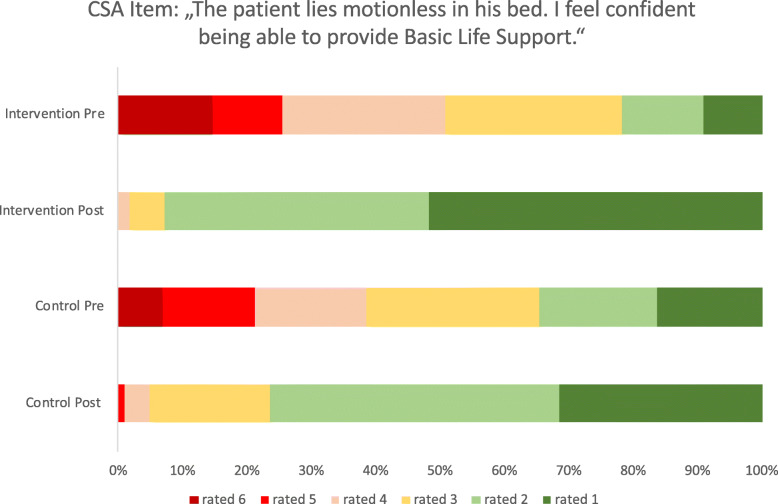
Descriptive analysis of learning gain pre and post interventional, graphics show the distribution of how confident the participants feel about the different advertised situations pre and post interventional. The evaluation offered 6 options from 1 = completely applies, 2 = mostly applies, 3 = still applies, 4 = rather does not apply, 5 = mostly does not apply, 6 = does not apply at all

The intervention group showed a significant greater learning gain in 6 out of 11 items (Table 2 and Fig. 4). For example item 3, “I feel confident to detect a cardiac arrest”, with a pre-rating of 5 (mostly does not apply) the intervention group achieved a learning gain of 3.21 points post-interventional, compared to 2.89 points in the control group (*p* = 0.007). This is a learning gain of 88.7% in the intervention group and 70.85% in control group (*p* = 0.014). Another two examples of items with a remarkable learning gain, are CSA-Items 2 and 10, with a learning gain of 86,65% and 81,13% in the intervention group, compared to 70,58% and 59,82% in the intervention group (Figs. 5, 6).

**Table 2:**
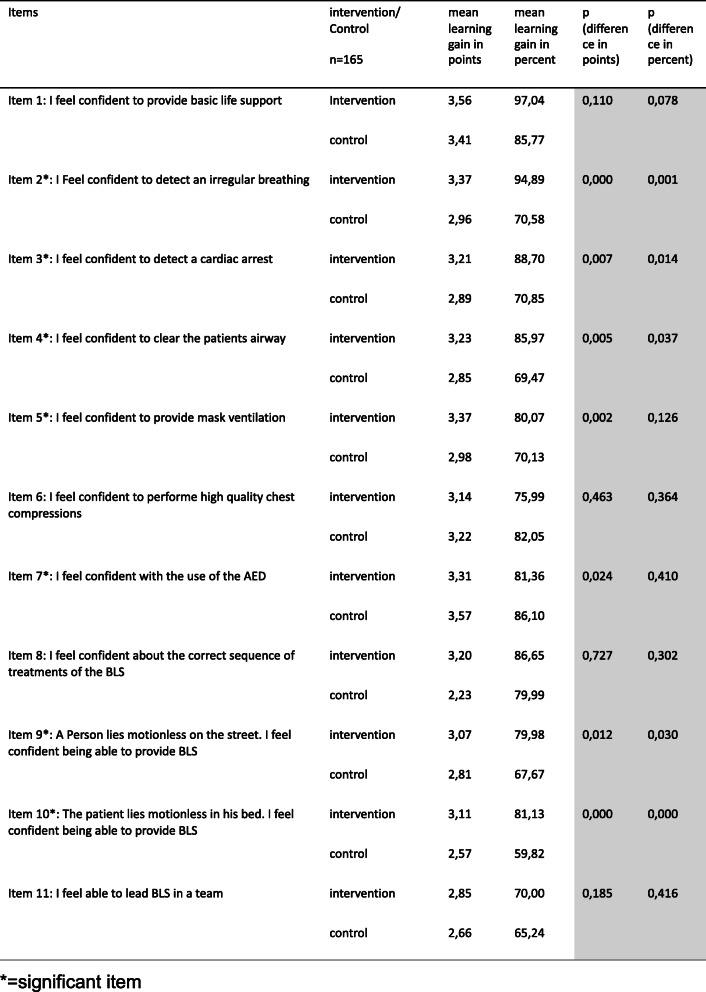
Learning gain with a pre-rating = 5 (mostly does not apply)

### SUS – system usability score

Fifty-three results of the SUS-questionnaire were analysed. The majority 96% (*n* = 51) would like to use this tool more frequently and felt very confident using the software. There were no relevant health problems like motion sickness while using the VR system (Supplement 3).

## Discussion

In this randomized, controlled study a BLS training with VR did not proof non-inferiority in comparison to a “classic” BLS training in respect of the primary outcome, the no flow time. However, the VR training was superior to classic training in 6 out of 11 items of the learning gain assessment (secondary outcome).

High quality chest compressions with a short no flow time are crucial for a good outcome after cardiac arrest. No flow time has to be as short as possible, however, there is no cut-off value above that a no flow time is not acceptable anymore. In this study, which aimed to proof non-inferiority of the VR group in comparison to the control group, we considered a deviation of 10% in no flow time as the limit for inferiority. In reality, both groups differed only by 11 s or 12%.

In 2008, Bobrow et al. showed that minimal interrupted chest-compressions are associated with an significant increase of survival after out-of-hospital cardiac arrest [15]. The no flow time or no-flow-fraction can be used as a sensitive parameter for quality of resuscitation, even though there are various influencing parameters such as giving rescue breaths or connecting the automated external defibrillator (AED). A review by Kramer-Johansen et al. concluded that in settings of ACLS (advanced cardiac life support), the no flow time before intubation should not exceed 15 s per minute in average [16], which would transfer to an overall no flow time of 45 s in the setting of our study. Assuming that 45 s are an acceptable no flow time, both groups of our study would have been significantly better. However, in the presented study, the time to detect the cardiac arrest was part of the no flow time, including the breath-check, emergency-call and installing the AED. The AED installation is a haptic process. The VR group had only used a virtual AED before taking the final examination. An integration of a real AED into the VR-Surrounding was not possible. So, its use was only simulated and had to be performed “in reality” in the final test for the first time. This might be an explanation for the slightly longer no flow time in the intervention group. This is supported by the CSA result of item 7 showing that the VR group felt to have problems using the AED. Further trainings in VR have to address this topic by combining a VR training with a training of haptic practical skills in the sense of an “hybrid lesson”.

The VR-Software used in this study has already an integrated feedback on chest compressions (heart rate, compression depth, correct hand position). This integrated feedback can increase the quality of the chest compressions in VR [17]. Further development of VR BLS-training has to include the no flow time into the feedback, to overcome its limitations. Then it can become a powerful tool not only for the education of medical students, but also for lay-rescuers.

The difference in no flow time discussed above might be of clinical significance, however, it is as important to have a first responder who is willing and confident to deliver chest compression. The VR group achieved a learning gain of 97.04% for the CSA item 1 (I feel confident to provide BLS), and a learning gain of 88.70% for the CSA item 3 (I feel confident to detect a cardiac arrest). So the VR training was associated with a significantly higher learning increase not only for the majority of items, but also for some crucial items.

There seems to be a good correlation between subjective learning gain in the CSA and objective learning gain in a summative assessment [18]. In this study, VR increased the learning gain for “emotional” items such as the self-confidence to deliver CPR, which is difficult to assess objectively. Furthermore, there was no difference in the quality of chest compressions between the two groups. The VR-Software used in this study has already an integrated feedback on chest compressions (heart rate, compression depth, correct hand position). Future Versions of CPR training in VR are supposed to include a demonstration and training of all technical skills with feedback.

The user-friendliness of VR was rated very high. VR is a novel teaching method many students in an increasing digital world feel confident with. Previous studies also demonstrated, that resuscitation training in VR is rated very positively [19, 20]. From a medical educative point of view it is very important to notice that in this study the participants were not only satisfied with VR teaching (high SUS), but also had high learning gain (CSA). This shows, that the VR module was teaching and not entertainment.

### Limitations

The secondary outcome parameter was the learning gain evaluated with the comparative self-assessment. As part of this method there is a group of participants with a pre-rating of one (approximately 10% in this cohort) who will not have a learning gain per definition. Accordingly, no conclusion can be drawn on how this group profited from the VR or the classic training in this study.

## Conclusion

CPR training using VR is a feasible and effective training method. However, for teaching technical skills, a classic training is still superior to a VR training. On the other hand, considering the overall learning gain, VR training is superior to the classic training in most items in a comparative self-assessment. Therefore, future BLS course-formats should consider the integration of VR technique into the classic CPR training or vice versa, to use the advantage of both teaching techniques.

## Supplementary Information

**Additional file 1.** CSA-Questionaire.

**Additional file 2.** System usability scale for VR-Soft-and Hardware.

**Additional file 3.** System Usability Scale Results (shortened version).

## Data Availability

The datasets analysed during this study are available from the corresponding author on reasonable request.

## Abbreviations

AED: Automated external defibrillator
BLS: Basic Life Support
CPR: Cardiopulmonary resuscitation
CSA: Comparative self-assessment
ERC: European Resuscitation Council
SUS: System usability score
VR: Virtual Reality

## Footnotes

1 https://www.vireed.de
