# Supplementary material for: Virtual reality as a teaching method for resuscitation training in undergraduate first year medical students: a randomized controlled trial
Source: Scand J Trauma Resusc Emerg Med. 2021 Feb 1;29:27. doi: 10.1186/s13049-021-00836-y (PMC7851931; doi:10.1186/s13049-021-00836-y)
Supplement: Supplementary file 2 — Additional file 2. System usability scale for VR-Soft-and Hardware. [file 13049_2021_836_MOESM2_ESM.docx]

Supplement 2: System usability scale for VR-Soft-and Hardware
