# Supplementary material for: Virtual reality as a teaching method for resuscitation training in undergraduate first year medical students: a randomized controlled trial
Source: Scand J Trauma Resusc Emerg Med. 2021 Feb 1;29:27. doi: 10.1186/s13049-021-00836-y (PMC7851931; doi:10.1186/s13049-021-00836-y)
Supplement: Supplementary file 3 — Additional file 3. System Usability Scale Results (shortened version). [file 13049_2021_836_MOESM3_ESM.docx]

Supplement 3: System Usability Scale Results (shortened version)
